# *Wolbachia* Density and Cytoplasmic Incompatibility in *Aedes albopictus*: Concerns with Using Artificial *Wolbachia* Infection as a Vector Suppression Tool

**DOI:** 10.1371/journal.pone.0121813

**Published:** 2015-03-26

**Authors:** Maurizio Calvitti, Francesca Marini, Angiola Desiderio, Arianna Puggioli, Riccardo Moretti

**Affiliations:** 1 Laboratory of Sustainable Management of the Agro-Ecosystem, ENEA − Italian National Agency for New Technologies, Energy and Sustainable Economic Development, Rome, Italy; 2 Laboratory of Parasitology and Entomological Surveillance, Istituto Zooprofilattico Sperimentale Lazio e Toscana, Rome, Italy; 3 Laboratory of Biotechnology, ENEA − Italian National Agency for New Technologies, Energy and Sustainable Economic Development, Rome, Italy; 4 Medical and Veterinary Entomology, Agriculture and Environment Centre “G. Nicoli”, Crevalcore, Bologna, Italy; International Atomic Energy Agency, AUSTRIA

## Abstract

The mosquito *Aedes albopictusi* is a competent vector of harmful human pathogens, including viruses causing dengue and chikungunya. Cytoplasmic incompatibility (CI) induced by endosymbiotic *Wolbachia* can be used to produce functionally sterile males that can be released in the field as a suppression tool against this mosquito. Because the available sexing methods are not efficient enough to avoid unintentional release of a few transinfected females, we assessed the CI pattern in crosses between *w*Pip *Wolbachia*-transinfected (AR*w*P) females and wild-type males of *Ae*. *albopictus* in this study. Quantitative polymerase chain reaction was used to monitor the titer of the *Wolbachia* strains that naturally infect *Ae*. *albopictus*, that is, *w*AlbA and *w*AlbB, in age-controlled males and females. Data were coupled with incompatibility level detected when the above-mentioned males were crossed with AR*w*P females. *Wolbachia* infection titer was also monitored in samples of wild caught males. Incompatibility level was positively correlated only with *w*AlbA density. Crosses between wild-type males having very low *w*AlbA density (<0.001 *w*AlbA/actin copy numbers) and AR*w*P females were partially fertile (CI_corr_ = 68.06 ± 6.20). Individuals with low *w*AlbA titer were frequently found among sampled wild males (30%–50% depending on the site and period). AR*w*P males can be as considered as a very promising tool for suppressing *Ae*. *albopictus*. However, crosses between wild males having low *w*AlbA density and AR*w*P females may be partially fertile. In the case of local establishment of the transinfected mosquito line, this occurrence may favor the replacement of the wild-type mosquitoes with the AR*w*P line, thus reducing the long-term efficacy of incompatible insect technique. Various alternative strategies have been discussed to prevent this risk and to exploit *Wolbachia* as a tool to control *Ae*. *albopictus*.

## Introduction

*Aedes* (*Stegomyia*) *albopictus* (Diptera: Culicidae), commonly known as Asian tiger mosquito, is one of the most invasive insect species worldwide [1,2]. It shows high vector competence for several arboviruses, including viruses causing dengue (DENV) and chikungunya (CHIKV) [3]. Although *Ae*. *albopictus* is not considered as the primary vector of DENV, it is becoming a major cause of viral outbreaks because of rapid changes in its overall distribution. In the last decade, *Ae*. *albopictus* caused new DENV epidemics in different countries such as Hawaii [4], Mauritius [5], and China [6]. With respect to CHIKV, a recent mutation in genes encoding envelope glycoproteins of CHIKV of an African lineage enhanced the adaptability of this virus to *Ae*. *albopictus* [7], leading to outbreaks in Indian Ocean islands in 2005–2006 [8] and in temperate regions such as Italy [9] and France [10]. The CHIKV mosquito-human transmission cycle recently has established in the Caribbean [11]. The associated epidemic is currently out of control, with more than 500,000 cases in a few months, and models are being developed to predict the possible spread of the virus in the Americas [12].

In recent years, interest in mosquito control strategies based on the principles of autocidal control, such as release of radio-sterilized males (Sterile Insect Technique, SIT) in field [13], has increased, thus providing new approaches by using innovative biotechnology (such as insect transgenesis and endosymbiont manipulation) [14–17]. The potential to combine these recent approaches with the classical SIT may offer viable solutions to overcome intrinsic limitations of the current conventional vector control strategies that mostly rely on insecticides and community participation. In addition to exerting negative effects on non-target insects and having toxicological impact on humans and environment, insecticides result in the establishment of resistant strains, as shown in some recent reports [18,19]. Therefore, alternative strategies to control mosquitoes are being sought worldwide.

Research on the endosymbiont *Wolbachia pipientis* (Alphaproteobacteria: Rickettsiales) has increased steadily in the last two decades, driven by the possibility of exploiting its biological properties as tools for insect pest and vector control [16,20]. This maternally inherited bacterium manipulates host reproduction and is carried by approximately 40% arthropod insect species as well as some crustaceans, mites, and filarial nematodes [21]. Cytoplasmic incompatibility (CI) is the most common reproductive phenotype observed in arthropod species infected with specific *Wolbachia* strains [,^22^–^27^]. First described in mosquito *Culex pipiens* [28–30], CI is a conditional embryonic lethality that occurs when males infected with CI-inducing *Wolbachia* strains are crossed with uninfected females (unidirectional CI [Uni-CI]) or with females carrying other incompatible *Wolbachia* strains (bidirectional CI [Bi-CI]).

In 2009, an incompatible *Ae*. *albopictus* strain (AR*w*P) was generated to support a SIT project against *Ae*. *albopictus* in Italy [31]. AR*w*P was obtained by replacing natural *Wolbachia* double infection with a single heterologous strain of *Wolbachia* (*w*Pip) taken from *Culex pipiens molestus* [32]. The transinfected *w*Pip strain was attributed to the *w*Pip-IV incompatibility group (Mylene Weill, pers. com.) according to a classification by Atyame *et al*. [33]. Infection parameters (maternal inheritance and fitness costs), male mating competitiveness performances, and CI features (induction of complete and not age-dependent CI when *w*Pip-transinfected males are crossed with wild-type females) were investigated both at laboratory level and on a semi-field scale [34,35]. The results of these studies were consistent with the traits needed to use the AR*w*P strain as the provider of ready-made sterility-inducer males for incompatible insect technique (IIT), which is an alternative autocidal approach based on the release of biologically incompatible males rather than irradiated males [15,36,37].

However, some major drawbacks have to be overcome, particularly against mosquito vector species, before IIT can be practiced in field. One of the major constraints is the inevitable co-release of females infected by a non-native *Wolbachia* strain that results in the release of incompatible males. This is because of the absence of an efficient sexing technology for the perfect separation of male and female pupae. In fact, at least 1% female contamination [38] is expected during the release of males. Model simulations based on laboratory experiments clearly predict that in cases where IIT strategy relies on a strong Uni-CI pattern (i.e., when the target population is uninfected), accidental co-release of *Wolbachia*-infected males and females may lead to an unwanted replacement of uninfected targeted population with the new infected population [39]. This would make IIT progressively ineffective. However, prediction becomes more complex when the target population harbors an incompatible *Wolbachia* infection type (Bi-CI), (as an example, if using the AR*w*P strain against the naturally infected *Ae*. *albopictus*). In this case, local coexistence of two different *Wolbachia* infection types may result in an unstable equilibrium that will evolve over time, resulting in the fixation of either one of the two infection types. According to Dobson *et al*. [39], this outcome would theoretically depend on two main factors: (i) pattern of CI (Uni-CI or Bi-CI) between the two populations and (ii) their competition both at larval and adult stages.

In this study, we provide new evidence on the first factor, i.e., CI pattern between naturally infected *Ae*. *albopictus* males (coinfected with *w*AlbA and *w*AlbB *Wolbachia* strains) and *w*Pip-transinfected AR*w*P females, that we recently proved to be partially bidirectional [34]. Crosses between AR*w*P females and wild-type males were partially fertile when wild-type males were aged more than two weeks. On one hand, this weakness in the reproductive barrier between wild-type and transinfected mosquitoes may be convenient because it allows us to easily outcross the AR*w*P line with wild-type populations, thus restoring genetic variability periodically. However, the possible effects of this in field should be carefully considered.

First, we considered fundamental to provide additional data on *w*AlbA and *w*AlbB density in wild type *Ae*. *albopictus* from two sites for comparison with previous reports [40,41]. Next, we coupled the molecular determination of *w*AlbA and *w*AlbB bacterial titers in age-controlled superinfected males with the CI level observed in their crosses with AR*w*P females.

At last, comparison of laboratory results with the outcomes of *Wolbachia* density monitoring performed on captured wild-type males has been discussed as a necessary step for developing a bio-ecologically safe, long-term, and area-wide suppression strategy against *Ae*. *albopictus* based on the exploitation of *Wolbachia*-induced CI.

## Materials and Methods

### Ethics Statement

Research performed on invertebrates such as mosquitoes does not require a specific permit according to the directive 2010/63/EU of the European Parliament and of the Council on the protection of animals used for scientific purposes. *Ae*. *albopictus* is not an endangered or protected species, and no specific permissions are needed for collecting its eggs or adults in Italy. Samples were not collected from private or protected areas (see below for locations). Two of the authors (MC and RM) voluntarily used their arms for blood feeding during the experiments. According to the ethics committee of ENEA, this practice is not considered human experimentation.

### Mosquito lines and rearing

This study included three *Ae*. *albopictus* populations: a *w*Pip-transinfected population (AR*w*P) [32] and two naturally superinfected populations (S_CRE_ and S_ANG_) obtained from eggs collected in North and Central Italy, respectively. The first collection site is an urban area in Crevalcore in Bologna province (CRE: 44°43′12.10″N, 11° 8′54.94″E) while the second collection site is in Anguillara Sabazia, a suburban area 25 km north of Rome (ANG: 42°5′29.32″N, 12°16′17.57″E). The three mosquito populations were reared as described below.

Larvae were brought to adulthood in 1.5-l larval trays, at a density of 5 larvae/ml. Larval food was provided as described in Sinkins *et al*. [42]. Adult mosquitoes were kept in 40 × 40 × 40-cm cages placed in a climatic chamber (T = 27 ± 2C°, RH = 70 ± 10%, L:D = 14:10 hours) and were fed with sucrose solution (10%) soaked on cotton.

### Age- and sex-specific *Wolbachia* density in S_CRE_ and S_ANG_ mosquito lines

Adult males and females from the two natural *Wolbachia*-infected lines were isolated at the emergence and were pooled to be aged in the following six age groups (1–3, 4–6, 7–9, 10–15, 16–20, and >20 days). Males and females from each mosquito line and age group were analyzed by quantitative polymerase chain reaction (qPCR; see below) to evaluate the variation in the mean density of each *Wolbachia* strain with aging.

### Crosses for CI strength assessment

Naturally infected males were crossed with 1-week-old AR*w*P females to determine eventual correlations between *w*AlbA and *w*AlbB densities and induced CI level. This study was planned regardless of the geographical origin; for this reason, only S_CRE_ males were used.

In all, 50 virgin *Ae*. *albopictus* males belonging to 3 different age groups (3 ± 1, 11 ± 1, and 19 ± 1 days) were singly placed in 20 × 10 × 5-cm mating-oviposition cages with a single 1-week old AR*w*P virgin female (AR*w*P ♀ × S_CRE_ ♂).

Because nuclear genes may be involved in generating incompatibility between populations [43], the AR*w*P strain was outcrossed for 5 generations with *Wolbachia*-cured S_CRE_ males before setting up the CI experiments.

After copulation, the males from incompatible crosses were stored in ethanol and frozen for subsequent qPCR to determine the titer of *Wolbachia* strains of naturally infected *Ae*. *albopictus*. Once mated, females were fed with blood on human arms and isolated. Eggs laid from each single female were collected on oviposition devices made of wet strips of crepe paper and were stored in an incubator (temperature, 27°C; RH, 90%) for 5 days. The percentage of hatched eggs was used to compare CI levels with those obtained using fertile control crosses. Females whose eggs did not hatch were dissected to determine whether their spermathecae were filled or not with spermatozoa and in the latter case were excluded from the analysis.

Concurrently, AR*w*P compatible crosses (AR*w*P ♀ × AR*w*P ♂) were set up using 1-week-old virgin females and males belonging to the three age groups mentioned above. Ten crosses were performed for males belonging to each age group to determine the background embryonic mortality unrelated to CI and to compute CI_corr_ index (see below). In addition, 20 1-week-old virgin females were mated with males aged 3 ± 1 days and were then fed with blood. After egg laying, qPCR analysis was performed to quantify *w*Pip *Wolbachia* titer and to ascertain whether *w*Pip density and fertility in AR*w*P females were correlated in the above laboratory conditions.

### *Wolbachia* density in wild caught males

*Wolbachia* titer of randomly captured wild males was surveyed at collection sites of the two studied populations (Crevalcore and Anguillara Sabazia in September 2013 and July 2014, respectively). Samples were collected using manual aspirators and by catching males flying around human operators. These *Wolbachia* density data were used to evaluate the risk of CI failure in crosses between wild-type males and AR*w*P females that were accidentally released or locally established after hypothetical IIT-based suppression programs.

### *Wolbachia* genotyping and qPCR

#### DNA purification and qPCR

Total DNA was extracted from the whole body of a single *Ae*. *albopictus* mosquito by using ZR Tissue & Insect DNA Kit MicroPrep (Zymo) according to manufacturer's instructions. Strain-specific primers were used to amplify *wsp*. The *w*AlbA-*wsp*, *w*AlbB-*wsp* and *w*Pip-*wsp* loci were amplified using previously described oligonucleotide primers pairs 328F/QArev2, 183F/QBrev2, *w*PF/*w*PR respectively, [34,40,44] to obtained 200-, 112- and 271-bp fragments, respectively.

Actin gene of *Ae*. *albopictus* was used as a nuclear reference and was amplified using primer pair actAlbqPCRsense (CCCACACAGTCCCCATCTAC) and actAlbqPCRantisense (CGAGTAGCCACGTTCAGTCA) to obtain a 119-bp amplification product.

Amplification reactions were performed using 20 μl of FluoCycle II SYBR Master Mix (Euroclone). Total DNA (2 μl) from each mosquito was used as a template for PCR and each reaction was performed in triplicate. PCR was performed in ABI Prism 7100 (Applied Biosystems) thermal cycler using the following amplification program: initial activation at 95°C for 5 min, followed by 40 cycles at 95°C for 15 s and 60°C for 1 min. Presence of specific amplification products was verified using dissociation curves.

#### Construction of plasmids for obtaining qPCR standard curves

Specific DNA sequences encoding *w*AlbA-*wsp*, *w*AlbB-*wsp*, *w*Pip-*wsp* and actin (quantitative reference) for qPCR amplification were cloned from the total DNA extracts. DNA fragments of *w*AlbA-*wsp* (382 bp) and *w*AlbB-*wsp* (501 bp) were amplified from the total DNA extracted from field-caught *Ae*. *albopictus* by using primer pairs 328F/691R and 183F/691R, respectively [44]. DNA extracted from *Cx*. *pipiens* was used as a template to amplify a *w*Pip-*wsp* gene fragment (404 bp) with primers 183F and *w*PR. All the amplicons were cloned in pCR 2.1 vector plasmid (TA Cloning Kit, Invitrogen).

The amplified sequences were assembled to obtain the plasmids pBS-A-B-act (containing *w*AlbA-*wsp*, *w*AlbB-*wsp* and actin gene fragments) and pBS-Pip-act (containing *w*Pip-*wsp* and actin gene fragments) by using the following procedure. Actin gene fragment was transferred from pCR 2.1 into *Bam*HI-*Not*I sites of pBluescript II SK (+) vector to produce pBS-act plasmid. Next, *w*AlbB-*wsp* and *w*Pip-*wsp* fragments were cloned from pCR 2.1 into *Not*I-*Sac*I sites of the pBS-act plasmid to produce pBS-B-act and pBS-Pip-act plasmids. Finally, *w*AlbA-*wsp* fragment was cloned from pCR 2.1 into *Kpn*I-*Xho*I sites of the pBS-B-act plasmid to produce pBS-A-B-act plasmid. All the obtained constructs were sequenced to assess the correct assembly and absence of unwanted sequence variations.

#### Statistical analysis and CI computation

Direct correlation between *Wolbachia* density (*w*AlbA and *w*AlbB) and mosquito age was investigated in both the sexes by using Spearman correlation test.

To test the correlation between CI level and *w*AlbA and *w*AlbB bacterial loads in males, a graph was plotted for *Wolbachia* density in each male used in each single-pair crossing experiment against observed CI expression.

Values of *Wolbachia* (*w*AlbA and *w*AlbB) density in each male used in incompatible crosses were grouped in *Wolbachia* density classes. For each cluster of *Wolbachia* density, we associated mean CI values found in crosses involving the corresponding males.

If the data set met the assumptions of normality (Shapiro–Wilk test, P > 0.05), one-way analysis of variance (ANOVA) was performed, followed by Bonferroni–Dunn multiple comparisons test. If the data set did not meet the assumptions of normality, non-parametric Kruskal–Wallis test was performed to determine whether this clustering highlighted significant differences between *Wolbachia* density in males and mean CI expressed in crosses. Dunn's test was used for pairwise comparison of mean *w*AlbA and *w*AlbB densities in males and CI values.

CI expression was calculated using egg mortality observed in each single-pair incompatible cross (AR*w*P ♀ × S_CRE_ ♂) and was compared with the mean hatching rate (mean ± SEM) observed in single-pair compatible crosses (e.g., AR*w*P ♀ × AR*w*P ♂) by using CI_corr_ index [45]. This index does not overestimate the CI level caused by other mortality factors such as mean embryonic mortality observed in compatible crosses and male age effects that generally decrease fertility. The formula used for calculating CI_corr_ index is as follows:

$$CI_{corr}\left(\%\right)=\frac{UnE_{CI}-UnE_{CC}}{100-UnE_{CC}}\times 100,$$

where *UnE*_*CI*_ is the proportion of eggs that did not hatch in crosses between different infection types and *UnE*_*CC*_ is the proportion of undeveloped embryos in compatible crosses. The value attributed to this last parameter was derived from AR*w*P compatible crosses performed using three classes of age-controlled males as described above.

All statistical analyses were performed using GraphPad Prism Software 6.0.1 version (GraphPad Software Inc., San Diego, CA, USA).

## Results

### Age- and sex-specific *w*AlbA and *w*AlbB densities

**Fig 1:**
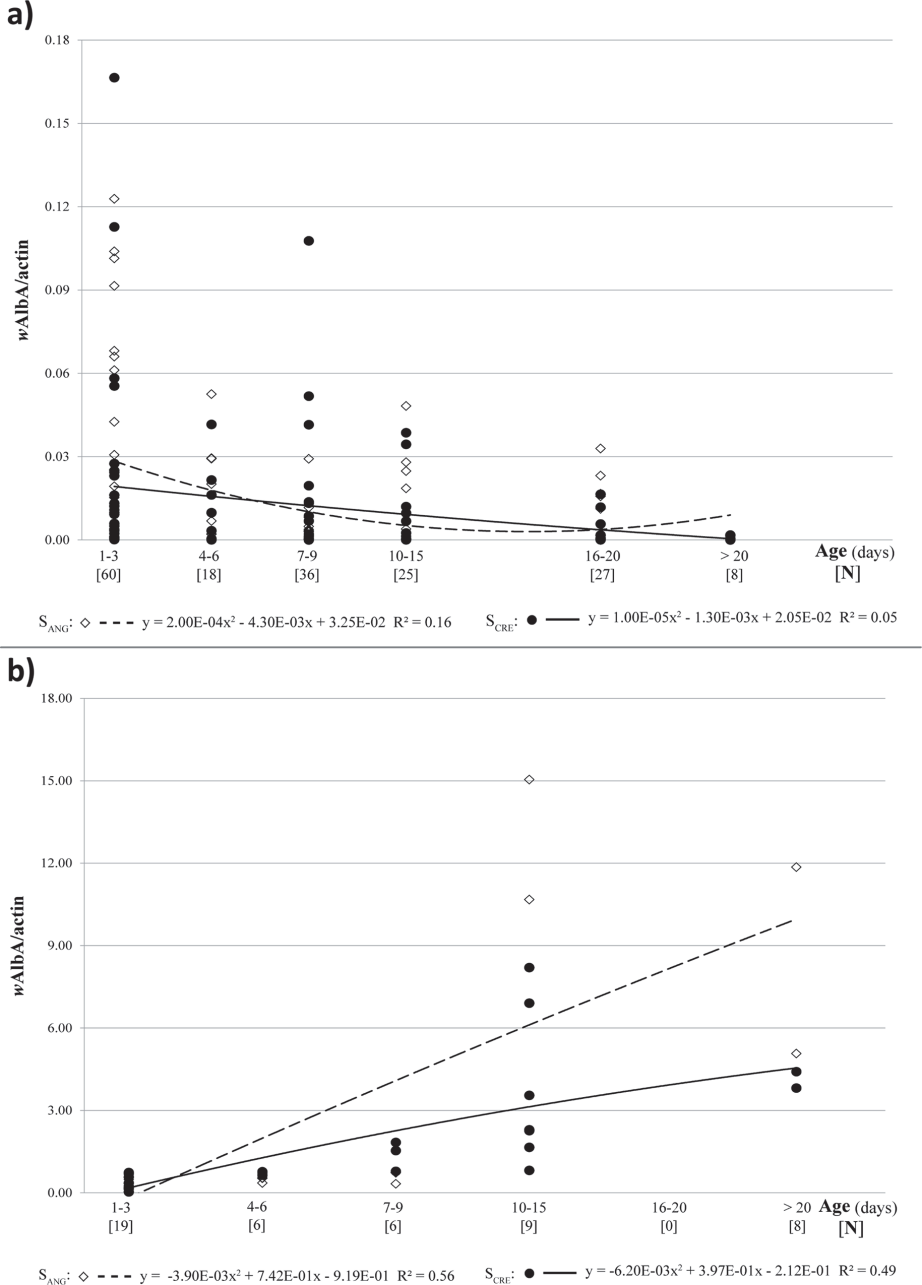
Age dependent variation of *w*AlbA density in *Ae*. *albopictus* males (a) and females (b) belonging to two Italian populations (S_ANG_ from Anguillara Sabazia, Rome: empty squares; S_CRE_ from Crevalcore, Bologna: full circles). Data trend is displayed via a polynomial trend-line together with the associated function (dashed line for S_ANG_, solid line for S_CRE_).

*Ae*. *albopictus* males showed remarkable individual variability in *w*AlbA density (Fig. 1A). In fact, *w*AlbA titers ranged from 0.0001–0.165 per actin copy number (*w*AlbA/actin). Complete loss of this *Wolbachia* strain was rarely observed in males (only 1 case out of 174); however, most males infected at low *w*AlbA densities (<0.001 *w*AlbA/actin) yielded negative results for standard PCR (S1 Fig.). Despite this high variability, we observed an evident reduction in the variation range with an increase in age. In fact, in young males (age, 1–3 days), *w*AlbA/actin ratios were widely different, ranging from <0.001 to >0.06 (mean ± SEM, 0.024 ± 0.005; N = 60). However, very low *w*AlbA levels (<0.001 *w*AlbA/actin) were detected more frequently in older males (age, >16 days), with a narrow range of variability (0.005 ± 0.002 *w*AlbA/actin; N = 35).

Despite the high individual variability, a significant negative correlation was observed between age of males and *w*AlbA density (Spearman r = −0.35; P ≤ 0.0001).

In general, S_CRE_ males were infected with lower mean *w*AlbA titer (0.010 ± 0.004 *w*AlbA/actin) than S_ANG_ males (0.020 ± 0.005 *w*AlbA/actin) irrespective of their age. However, this difference was not statically significant (ANOVA, F_(1,94)_ = 1.28; P > 0.05).

In contrast to the trend observed in males, *w*AlbA density was positively correlated with age in females (Spearman r = 0.92, P ≤ 0.0001; Fig. 1B). The highest average *w*AlbA titer was observed in S_ANG_ females (2.420 ± 1.005 *w*AlbA/actin) and not in S_CRE_ females (1.800 ± 0.680 *w*AlbA/actin); however, data were not sufficiently robust to highlight a statistically significant difference with respect to the geographical origin of the females (ANOVA, F_(1,45)_ = 0.15; P > 0.05).

**Fig 2:**
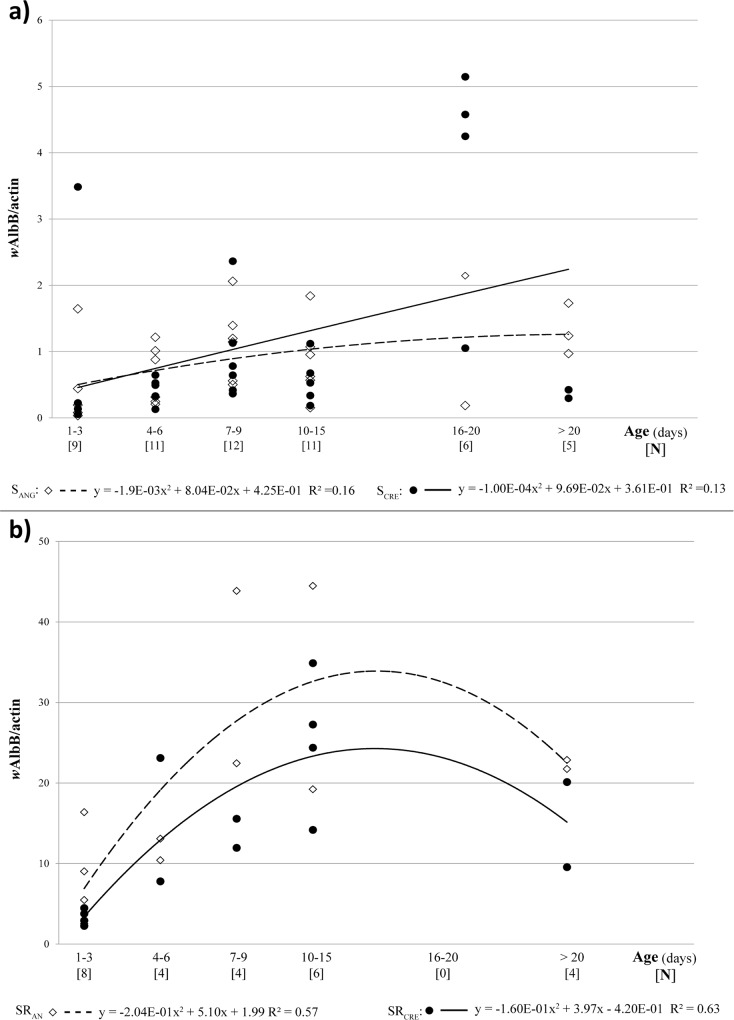
Age dependent variation of *w*AlbB density in *Ae*. *albopictus* males (a) and females (b) belonging to two Italian populations (S_ANG_ from Anguillara Sabazia, Rome: empty squares; S_CRE_ from Crevalcore, Bologna: full circles). Data trend is displayed via a polynomial trend-line together with the associated function (dashed line for S_ANG_, solid line for S_CRE_).

We confirmed that *w*AlbB was more abundant than *w*AlbA (*t*-test, P < 0.0001; Fig. 2). On an average, *w*AlbB was 40–50- and 7–9-fold more concentrated than *w*AlbA in males and females, respectively. In *Ae*. *albopictus* males (Fig. 2A), mean *w*AlbB titer increased with age (Spearman r = 0.38, P = 0.002). On the other hand, *w*AlbB density increased in females aged 10–15 days (Spearman r = 0.69; P ≤ 0.0001) and declined in older females (age, 19–21 days; Fig. 2B). Like *w*AlbA, *w*AlbB was more abundant on an average in both the sexes of the S_ANG_ population (0.80 ± 0.10 *w*AlbB/actin in males; 19.28 ± 3.76 *w*AlbB/actin in females) than in those of the S_CRE_ population (0.52 ± 0.16 *w*AlbB/actin in males; 14.00 ± 2.81 *w*AlbB/actin in females). However, the differences were not statistically significant (ANOVA, F_(1,24)_ = 1.09; P > 0.05).

### Effect of male *w*AlbA and *w*AlbB densities on CI expression

A general trend of significant decrease in the percentage of egg hatching related to male aging was observed in compatible AR*w*P ♀ × AR*w*P ♂ crosses by using 1-week-old females. In fact, mean percentages of egg hatching were 74.51 ± 4.89, 65.62 ± 3.22, and 49.10 ± 5.88 when males were 3 ± 1, 11 ± 1, and 19 ± 1 days old, respectively. Particularly, the oldest males were significantly less fertile than younger males (ANOVA, F_(3,36)_ = 9,419; P < 0.0001).

**Fig 3:**
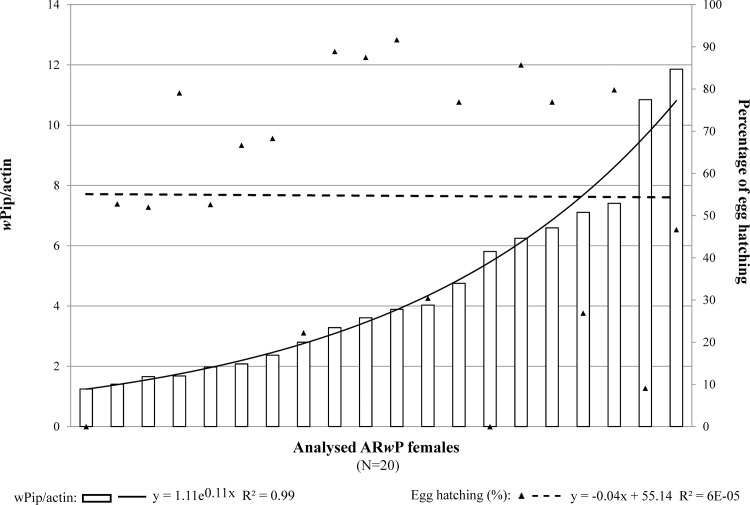
Correlation between percentage of egg hatching in AR*w*P × AR*w*P compatible crosses and *w*Pip *Wolbachia* density in females. Data trend is displayed via a polynomial trend-line together with the associated function.

Results of qPCR showed that fertility in compatible crosses was not correlated to *w*Pip density in AR*w*P females (Spearman r = 0.012; P = 0.958; Fig. 3). Findings allowed us to exclude *w*Pip titer as a factor determining egg mortality under the tested experimental conditions.

**Fig 4:**
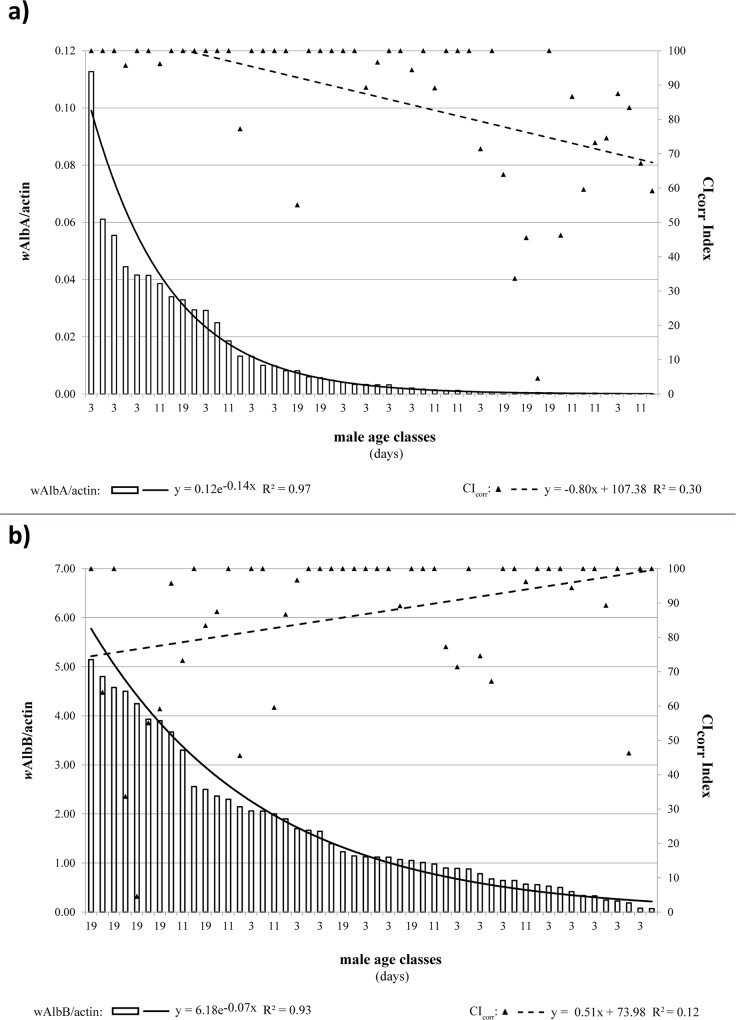
Correlation between *w*AlbA (a) or *w*AlbB (b) *Wolbachia* density and expressed CI levels (CI_corr_ index) in *Ae*. *albopictus* males when crossed with AR*w*P females at three different ages (3 ± 1; 11 ± 1; 19 ± 1). Data trend is displayed via a polynomial trend-line together with the associated function.

Results of qPCR analysis relative to *w*AlbA and *w*AlbB densities in males from incompatible crosses are reported in Fig. 4 along with the observed CI levels (CI_corr_ index). Our results showed that induction of strong CI (CI_corr_ index ≅ 100) corresponded to males with *w*AlbA titers ranging from 0.01 to 0.11 *w*AlbA/actin (Fig. 4A). It is worth highlighting that almost all crosses showing incomplete CI (<80%) involved older males and some young males apparently harboring low *w*AlbA density (<0.001 *w*AlbA/actin) since emergence. Furthermore, old males with relatively high *w*AlbA titers (>0.01 *w*AlbA/actin) induced complete or approximately 100% CI.

Male *w*AlbA densities were clustered into three classes <0.001, 0.001−0.010, and >0.010 *w*AlbA/actin, and their related mean CI levels were calculated (Table 1). The average CI level (CI_corr_ = 68.06 ± 6.20) induced by males with lowest *w*AlbA density was significantly lower than that in other classes (Kruskal–Wallis and Dunn multicomparison test P < 0.05).

**Table 1:** Density of *w*AlbA *Wolbachia* and associated CI level expressed by *Ae*. *albopictus* males when crossed with AR*w*P females.

***w*AlbA density classes**(*w*AlbA/actin)	**N**	**CI**_corr_(mean ± SEM)
<0.001	17	68.06 ± 6.20*
0.001−0.010	17	95.57 ± 2.68
>0.010	16	98.08 ± 1.43

Density values have been clustered in three density classes.

* − statistically significant difference, by Bonferroni’s multiple comparison test with α = 0.05.

**Fig 5:**
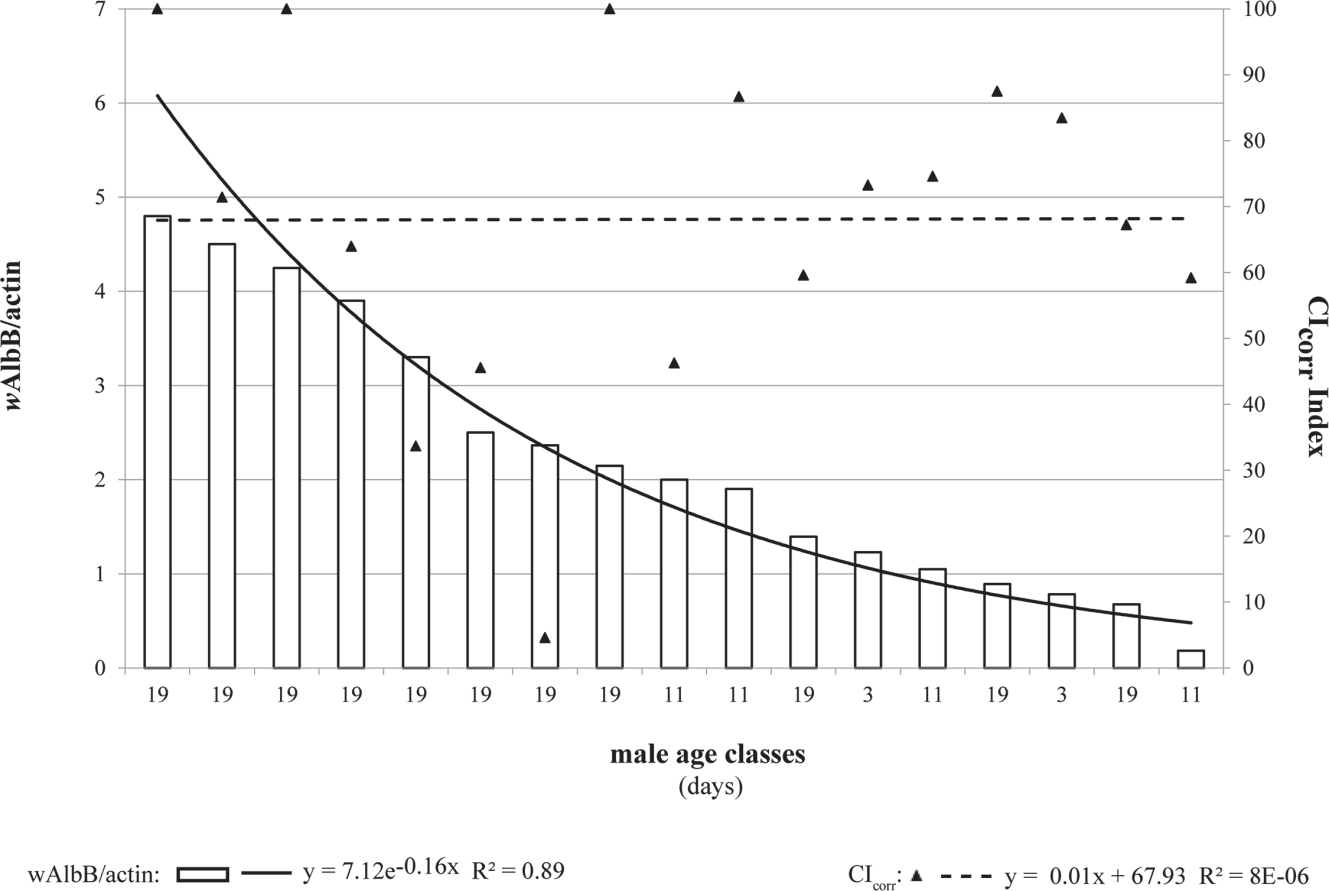
Correlation between *w*AlbB density and expressed CI level (CI_corr_ index) in *Ae*. *albopictus* males characterized by very low *w*AlbA titer when crossed with AR*w*P females. Data trend is displayed via a polynomial trend-line together with the associated function.

In contrast, male *w*AlbB titer showed no apparent correlation with CI penetrance in crosses involving AR*w*P females (Fig. 4B). To evaluate the specific role of *w*AlbB as a CI inducer, we restricted the correlation analysis to cases in which *w*AlbA density was low (<0.001 *w*AlbA/actin). By following this method, no significant correlation was observed between *w*AlbB concentration and CI expression (Fig. 5).

### *Wolbachia* density in wild caught males

**Fig 6:**
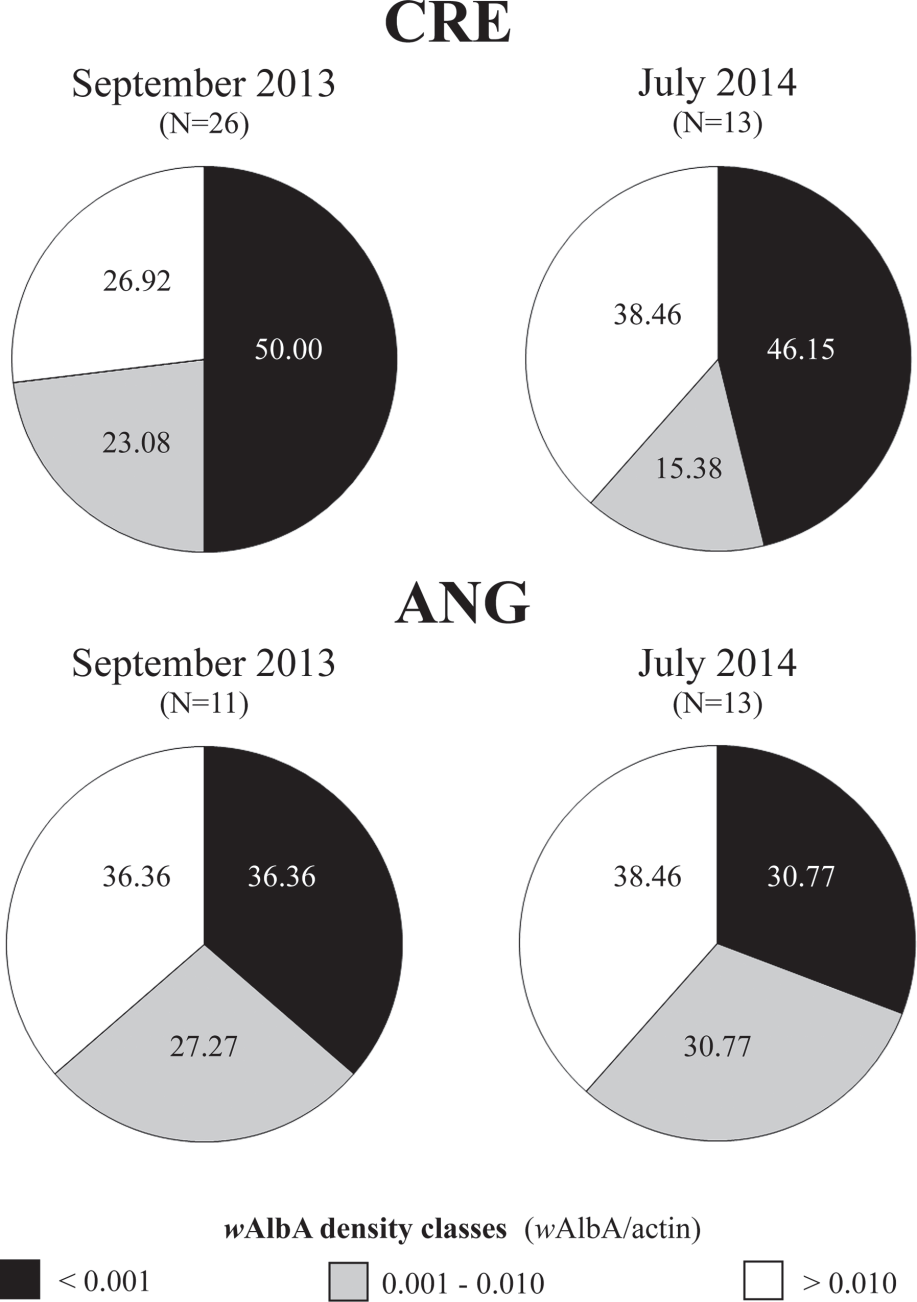
Density of *w*AlbA *Wolbachia* in wild caught *Ae*. *albopictus* males from two Italian sites (Crevalcore, CRE, Bologna; Anguillara Sabazia, ANG, Rome) and two periods (September 2013; July 2014). Density values have been clustered in three density classes. The individuals belonging to each density class are reported as percentages of the whole amount. CI level is expected to decrease to about 68% in crosses between AR*w*P females and males with *w*AlbA density values <0.001 *w*AlbA/act.

Results of qPCR analysis of wild collected *Ae*. *albopictus* are reported in Fig. 6 based on the same *w*AlbA density classes defined in the previous experiment. In September 2013, approximately 50% males collected from Crevalcore were infected with very low *w*AlbA titers (0.0001−0.001 *w*AlbA/actin). The males belonging to the above density class decreased to 46.15% in samples collected in July 2014 in favor of males infected with higher *w*AlbA titers (>0.01 *w*AlbA/actin). Among *Ae*. *albopictus* males collected from Anguillara Sabazia, proportion of males with infection titers below the lowest *Wolbachia* density class (0.0001−0.001 *w*AlbA/actin) ranged between 36.36% in September 2013 and 30.77% in July 2014.

Overall, males infected with *w*AlbA titers compatible with the expected complete or almost complete CI (>0.001 *w*AlbA/actin) ranged from 50.00% to 69.23% depending on the site and period.

## Discussion

Various strategies exploiting *Wolbachia* infection are currently being investigated for vector control [16]. Of these, *Wolbachia*-induced CI has been proposed as a method to produce functionally sterile males that can be released in field for vector suppression, in accordance with the principles of SIT [13,14]. *Wolbachia* transinfection techniques have been used to establish new laboratory lines of important vector species to obtain biological traits suitable for this purpose [46]. Specifically, CI relationships with wild-type populations, male competitiveness in comparison with wild-type males, and fitness parameters contributing to efficient mass rearing should be studied carefully.

Nearly five years after its generation, the *w*Pip-transfected line of *Ae*. *albopictus* (AR*w*P) is presently being evaluated for developing the most appropriate CI-based suppression strategy against this mosquito species. In previous studies, we have ascertained that AR*w*P line shows some desirable traits of an ideal IIT effector against *Ae*. *albopictus* (full CI, male mating competitiveness and female fitness not significantly different from wild-type *Ae*. *albopictus*) [32,34,35]. However, we also found that although CI between AR*w*P males and wild-type females was always complete, naturally infected males were not equally strong CI inducers towards AR*w*P females [34]. Moreover, although wild-type males were coinfected with *w*AlbA and *w*AlbB *Wolbachia* strains, only *w*AlbA strain determined a pattern of complete bidirectional incompatibility with *w*Pip-infected females. In addition, male aging seemed to be the factor responsible for the reduction in CI level [34]. In this study, we analyzed *w*AlbA and *w*AlbB titers in age-controlled males collected from two sites and the associated CI levels after crossing these males with AR*w*P females. In fact, establishment of a correlation between *Wolbachia* density and CI would help in evaluating the risk of bidirectional CI failure by sampling wild-type males before hypothetical field releases.

Results of qPCR supported the results of previous studies [40,41], confirming that *w*AlbA is constantly maintained at a lower density than *w*AlbB both in males and females. In addition, density patterns of both the *Wolbachia* strains were strongly dependent upon sex, with females showing the highest densities and males exhibiting a dramatic decrease in *w*AlbA titers with age. Although we clearly confirmed that adult age was a fundamental factor influencing the density of both the bacterial strains, we realized that in general, the density of *Wolbachia* strains in naturally infected *Ae*. *albopictus* was an unpredictable individual feature that was partially related to the geographical origin of the population [40] and to environmental conditions (temperature and food availability) in which the larvae developed [42].

Differences observed at the population level were not significant in our study and did not involve the general trend of infection dynamics but confirmed that bacterial load may vary locally and depending on the period of the year. In fact, larger differences between populations have been observed in previous studies investigating *w*AlbA and *w*AlbB densities in *Ae*. *albopictus* populations from Reunion island, Greece, and Corsica [40].

Correlation studies between CI and *Wolbachia* density have shown that incompatibility level is positively related to male *w*AlbA density. However, this correlation is not linear. In fact, only males with very low *w*AlbA titers (<0.0010 *w*AlbA/actin) induced significantly lower levels of CI while those with higher *w*AlbA density did not show CI weakening. These findings relative to *w*AlbA partially agree with the model proposed by Breeuwer and Werren [47], which states that decrease in CI penetrance in old males is directly proportional to *Wolbachia* density in the testes or sperm cysts in general [45,48–56]. Notably, a marked decrease in incompatibility level was previously observed when 10-day-old *w*AlbA mono-infected males were crossed with *Wolbachia*-free females [41].

In contrast, *w*AlbB, the most abundant *Wolbachia* strain in superinfected males, did not show any correlation with the observed CI in crosses with AR*w*P females. In fact, the observed decrease in the CI level countered the initial increase in *w*AlbB density in aging males, which was possibly mediated by a concurrent decrease in *w*AlbA density. Moreover, in males with very low *w*AlbA titers, CI expression did not change in response to changes in *w*AlbB density. These results agree with those of a previous study, which showed that crosses between AR*w*P females and *w*AlbB or *w*AlbA mono-infected males were approximately 20% fertile and completely unfertile, respectively [34].

Previous studies have shown that *Wolbachia* infected various host tissues but was mostly (at least 20 folds more) concentrated in *Ae*. *albopictus* gonads [57]. Particularly, in Koh Samui and Mauritius strains, mono-infected with *w*AlbA, *Wolbachia* cannot be detected in non-reproductive tissues [58]. In addition, *w*AlbA and *w*AlbB strains showed a highly similar tissue tropism [57,58]. Thus, although our data concerned total body molecular quantification, we can confirm that *w*AlbA plays the main role in determining the complete bidirectionality in CI pattern occurring between native and AR*w*P *Ae*. *albopictus*. Under these premises, ascertaining *w*AlbA titer in wild collected males is crucial because changing environmental conditions may cause *Wolbachia* density to differ significantly. In fact, analysis of males from Crevalcore and Anguillara sites showed very low *w*AlbA titer in almost half of all the males (on average 45% falling in <0.0001 *w*AlbA/actin density group), which was in accordance with previous field data [40]. This means that AR*w*P females could be fertile not only with AR*w*P males but also (at least partially) with some wild-type males, presumably gaining a reproductive advantage compared with wild-type females.

Therefore, the unintentional but repeated release of AR*w*P females during IIT should be approached with caution for a series of reasons.

First, the lack of a sexing system that guarantees the total absence of females in the released population increases the vulnerability of any autocidal application against vector mosquitoes because released females can blood feed and transmit diseases [13,15]. This vulnerability further increases when we apply the IIT strategy because accidentally released females can mate with the released males and reproduce. In the specific case of the AR*w*P strain, the weakness of the bi-CI, that we have highlighted in this work, must be considered as further factor affecting the IIT failsafe.

On the other hand, based on the modeling approach [39,59], presence of two reciprocally completely incompatible mosquito populations at one site is desirable because the competition decreases the number of biting females expected in the long-term for a single population. In addition, a recent paper reported about the stable coexistence in a site of two molecular *Wolbachia* strains of *Culex pipiens*, characterized, like AR*w*P and wild *Ae*. *albopictus*, by partially bidirectional CI relationships [60] However, the outcome of a partial failure in the bidirectionality of the CI pattern between AR*w*P and wild-type *Ae*. *albopictus* has not yet been properly investigated by experimental validation of specific theoretical models.

For the reasons above stated, awaiting for the advent of a very efficient sexing system, other strategies could be developed to exploit the favorable properties of the AR*w*P strain.

An advancement of the IIT-based suppression strategy could be the concurrent or sequential release of males belonging to two reciprocally incompatible lines. This approach could drastically reduce the possibility of establishing a transinfected line because of incompatible crosses that would occur in most cases [15]; however, this should be experimentally validated. Suitable *Ae*. *albopictus* lines are already available [61,62]; nevertheless, other lines can be specifically established for this purpose.

Furthermore, an alternative strategy using a combination of IIT and SIT involving irradiation of incompatible males at radiation doses just high enough to induce sterility in any females that are not removed but not sufficient to affect male fitness should be considered [16,63,64]. This approach is expected to be promising if, life most insect species, *Ae*. *albopictus* females are more radiosensitive than males [65]. An important advantage of this modified IIT strategy over the traditional SIT strategy would be the absence of any residual male fertility, necessarily tolerated by the latter strategy to save male mating competitiveness [66,67].

As long as a method to release large amounts of only males is available and/or specific population dynamic models are experimentally validated, the latter strategy seems to be the easiest to test and propose. Overall, the goal of field application of the AR*w*P line to control *Ae*. *albopictus* based on IIT/SIT approaches seems achievable. However, as shown in this study, a series of necessary steps need to be considered to ensure that the application strategy is effective and safe in the long-term.

## Supporting Information

S1 Fig Evaluation of PCR sensitivity in relation to increasing *w*AlbA titers, calculated as *w*AlbA/actin copy numbers by qPCR on independent mosquito extracts: (1) 0.0001; (2) 0.0010; (3) 0.0039; (4) 0.0108; (5) 0.0582; (6) 0.1671; (7) 2.2938; (8) no template PCR control. For *wsp*A gene amplification 328F and 691R oligonucleotides were used, according to the following PCR program: 95°C for 3 min; then 35 cycles at 95°C for 30 s, 55°C for 30 s and 72°C for 35 s; finally an elongation step at 72°C for 7 min. The expected *wsp*A amplicon was of 382 bp.
(TIF)
