# Supplementary figures and images for: Wolbachia Density and Cytoplasmic Incompatibility in Aedes albopictus: Concerns with Using Artificial Wolbachia Infection as a Vector Suppression Tool
Source: PLoS One. 2015 Mar 26;10(3):e0121813. doi: 10.1371/journal.pone.0121813 (PMC4374832; doi:10.1371/journal.pone.0121813)

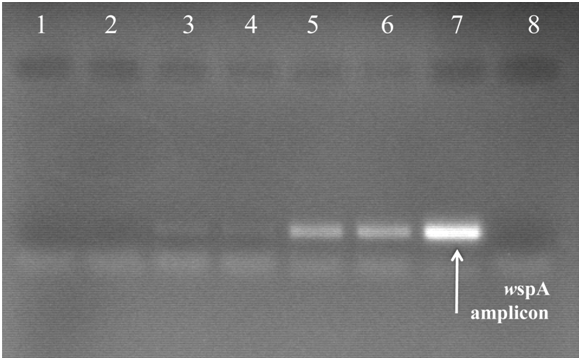

Supplement: S1 Fig — For wspA gene amplification 328F and 691R oligonucleotides were used, according to the following PCR program: 95°C for 3 min; then 35 cycles at 95°C for 30 s, 55°C for 30 s and 72°C for 35 s; finally an elongation step at 72°C for 7 min. The expected wspA amplicon was of 382 bp. (TIF) [file pone.0121813.s001.tif]
